# Thymic epithelial tumors: Do we know all the prognostic factors?

**DOI:** 10.1111/1759-7714.13750

**Published:** 2021-01-02

**Authors:** Magdalena Knetki‐Wróblewska, Dariusz M. Kowalski, Marta Olszyna‐Serementa, Maciej Krzakowski, Małgorzata Szołkowska

**Affiliations:** ^1^ Department of Lung Cancer and Chest Tumours Maria Skłodowska‐Curie National Research Institute of Oncology Warsaw Poland; ^2^ Department of Pathology National Tuberculosis and Lung Diseases Research Institute Warsaw Poland

**Keywords:** Prognostic factors, survival, thymic epithelial tumors

## Abstract

**Background:**

Thymic epithelial tumors constitute a morphologically and clinically diverse group of rare neoplasm of the anterior mediastinum.

**Methods:**

Here, we present an analysis of 188 patients diagnosed with primary thymic tumors between 1995 and 2015. The prognostic value of selected clinical and morphological factors was assessed in relation to overall survival and recurrence‐free survival.

**Results:**

The risk of recurrence increased significantly in thymic carcinoma diagnosis (*P* = 0.0036), co‐occurrence of other diseases, and weight loss (*P* = 0.0012 and 0.0348, respectively). Multivariate analysis showed that the most important independent risk factor for disease recurrence was clinical stage IV (*P* = 0.0036). A total of 63 patients (33.5%) died. In the univariate analysis, the following factors were considered as independent prognostic factors for overall survival: clinical stage (*P* < 0.0001), histological type (*P* < 0.0001), lymph node involvement (*P* < 0.001), WHO performance status 2 (*P* < 0.0001), anemia (Hb <9.5 g/dL; *P* = 0.0002), leucocytosis (>12.5 G/L; *P* = 0.0011), LDH level (>185 U/L; *P* < 0.0001), concomitant diseases (*P* = 0.0012) and weight loss (*P* < 0.0001).The strongest independent risk factor for death was stage IV disease (*P* < 0.001).

**Conclusions:**

The results confirmed a fairly good prognosis for patients with thymic epithelial tumors. Clinical stage was the most important prognostic factor, but, some additional clinical factors may also have prognostic value.

## Graphical abstract

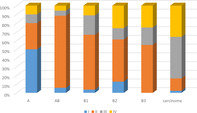

We present an analysis of 188 patients diagnosed with primary thymic tumours. The prognostic value of selected clinical and morphological factors was assessed in relation to survival. Multivariate analysis showed that the most important independent risk factor was CS IV. The risk of recurrence increased significantly in thymic carcinoma, comorbidities occurrence and weight loss. Some additional clinical factors including LDH elevation, leucocytosis, anemia may have prognostic value.

## Introduction

Thymic epithelial tumors (TETs) belong to the group of rare neoplasms and account for 0.05% of all malignant tumors.^1^, ^2^ TETs are the most common mediastinal tumors.^3^ Most TETs are thymomas with different subtypes with a good prognosis globally. Between 10% and 20% of all thymic epithelial malignancies are thymic carcinomas, most commonly of squamous cell histology (80% of cases).^4^

TETs most commonly affect people between the ages of 40 and 60, with a similar frequency among female and male. One third of patients are asymptomatic, and the tumor is discovered accidentally. In other cases, respiratory symptoms and less common systemic symptoms are present.^5^ Some patients have paraneoplastic syndromes, most often myasthenia gravis.

In the majority of cases, TETs are diagnosed as locally advanced, involving only the thymus, with the possible infiltration of adjacent structures. In a small percentage of patients (below 10%), the disease is generalized at the time of diagnosis.^5^

The results of previously published studies indicate that independent prognostic factors for overall survival (OS) among patients with TETs, including thymic carcinomas, include age, tumor size, histological type according to the WHO classification, clinical stage (CS) according to the Masaoka, type of neoadjuvant treatment, presence of paraneoplastic symptoms, lymph node involvement and completeness of surgical resection.^3^, ^5^, ^6^, ^7^, ^8^ Additionally, resection radicality, clinical stage and histological type are, together with the initial tumor size, the risk factors for disease recurrence after surgery.^9^ There have also been reports of the prognostic value of the so‐called circulating biomarkers (C Reactive Protein – CRP and lactate dehydrogenase – LDH),^10^ as well as the importance of the neutrophil‐to‐lymphocyte ratio (NLR)^11^ for survival and prognosis.

The aim of this study was to determine the prognostic value of selected clinical and laboratory parameters in patients with TETs in relation to survival.

## Methods

### Patient characteristics

A retrospective analysis of data from 188 patients (92 – male; 96 – female) treated at the Department of Lung Cancer and Chest Tumors, Maria Sklodowska‐Curie National Research Institute of Oncology in Poland and at National Tuberculosis and Lung Diseases Research Institute from 1995 to 2015, was carried out. The median age of the total population was 54.3 years (male – 51.7, female – 56.9). The clinical information was obtained from patients' medical records. The baseline demographic and clinical characteristics of patients are shown in Table 1.

**Table 1:** Baseline demographic and clinical characteristics of the study group

Characteristic	Total	%	Men	%	Women	%	*P*‐value
Total number of patients, n	188		92	48.7	96	51.3	
Age (years)	54.3 (16–84)		51.7		56.9		**0.009**
Symptoms of disease	110	58.5	57	6.6	53	55.2	0.3
Myasthenia	47	25	26	28.2	21	22.0	0.37
WHO performance status							
0 1 2 Missing data	54 120 10 4	29 65.2 5.4	27 57 6	29 64 6.7	28 63 4	30 66.3 4.2	0.74
Concomitant diseases^†^	105	55.8	46	50	59	61.5	0.13
Other neoplasms	19	10	9	9.7	13	7.0	
Other autoimmune diseases	18	9.5	6	6.5	12	12.5	0.17
Weight loss >10%	38	20	18	19.5	20	20.8	
Hb concentration (g/dL)	12.51 ± 2.21		12.87 ± 2.62		12.19 ± 2.6		0.03
LDH activity (IU/L)	170 (150–200)		165 (146–200)		170 (150–220)		0.17
Masaoka clinical stage							
I II III IV	14 99 42 33	7.5 53 22.5 17	6 48 21 17	6.6 52.2 22.8 18.4	8 51 21 16	8.4 53.1 21.9 16.6	0.95
Histological type according to the WHO classification^‡^							
A AB B1 B2 B3 Thymic carcinoma^§^ Unspecified/other	10 52 26 31 20 42 6	5.3 27.6 13.8 16.5 10.6 22.3 3.2					
Site of metastases Pleura Lungs Lymph nodes Bones Liver Central nervous system Other	20 7 25 3 3 1 2	10 3.7 13 1.5 1.5 <1 <1					

^†^ Hypertension (*n* = 96; 51.1%), coronary artery disease (*n* = 35; 18.6%), chronic obstructive pulmonary disease (*n* = 23; 12.2%), diabetes (*n* = 9; 4.7%), kidney failure (*n* = 4; 2.1%).

^‡^ Patients with tumor type assessed according to previous classifications (primarily according to the Muller‐Hermelink classification; medullary, mixed, organoid, cortical, well‐differentiated thymic cancer^12^) were assigned to one of the WHO categories, based on the analysis of the pathological description.

^§^ Included five patients with carcinoid.

The clinical stage was determined based on the results of imaging tests performed before the decision on treatment was made (mainly chest computed tomography [CT]; less frequently MR. To ultimately determine the disease stage, descriptions of pathological examination of the material obtained during thymectomy were analyzed.

The stage was determined according to the Masaoka classification.^13^ In a small percentage of patients for whom the Masaoka‐Koga classification (since 2015)^14^ was used, the disease stage was reclassified according to the Masaoka criteria, based on the result of pathological examination. Reclassification all patients according the Masaoka‐Koga classification was not possible due to the retrospective nature of the work and limited availability of archival samples. In patients diagnosed with thymic carcinoma, the clinical stage was assessed according to the simplified TNM system.^4^

The tumor histological type was assessed according to the WHO 2004 classification (A, AB, B1, B2, B3, thymic carcinoma).^12^ Patients with tumor type assessed according to previous classifications (primarily the Muller‐Hermelink classification) were assigned to one of the WHO categories based on the analysis of the pathological description.

In patients receiving neoadjuvant or palliative chemotherapy, the response was assessed according to the Response Evaluation Criteria in Solid Tumors (RECIST) criteria, version 1.1.^15^

### Statistical analysis

Categorical variables are presented in the form of the absolute and relative frequency of distinguished units, and the results of quantitative variables as arithmetic means, with standard deviation in the case of normal distributions or medians and quartiles (lower and upper) in the case of irregular distributions. To compare the incidence of individual variants of qualitative variables, the chi‐square test or Fisher's exact test was used. The equality of the average values of the two groups was verified by Student's *t*‐test or Mann‐Whitney test (irregular distributions), and in the case of three or more groups, the parametric or nonparametric analysis of variance was used, with appropriate tests after the fact.

After the estimation of survival function by the Kaplan‐Meier method, the homogeneity of the variables in different subgroups was verified by log‐rank test.

The prognostic value of selected variables (indicated in multivariate analyses as affecting survival with *P* < 0.1) was assessed using a multivariate Cox proportional risk analysis.

A verification of null hypotheses was carried out at the level of statistical significance alpha <0.05. Two‐sided testing was used.

A statistical analysis was performed using the SAS 9.2 statistical package (SAS Institute, US).

## Results

### Patients and interventions

Among 188 patients treated from January 1995 to December 2015 the most common types were thymomas AB (*n* = 52; 28%) and thymic carcinomas (*n* = 42; 23%).

**Figure 1:**
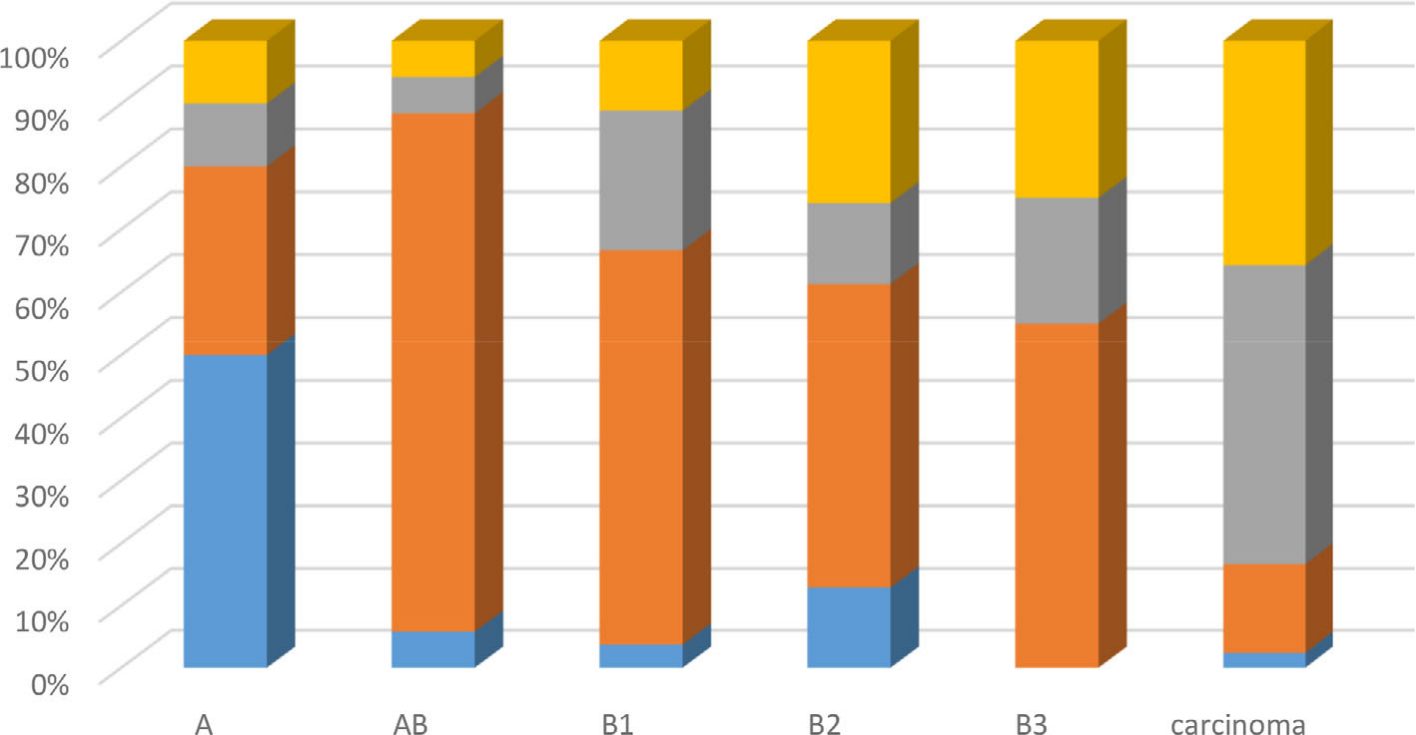
Distribution of histological subtype and clinical stage (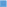) I, (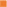) II, (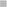) III, (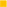) IV.

Stage II was the most common (52.5% of patients), while stages I, III and IV occurred in 7.5%, 22.5% and 12.5% of patients, respectively. Thymic carcinomas were most frequent in patients with stage III and IV disease, while A and AB types were most commonly diagnosed in stages I and II, respectively (Fig 1).

Surgery was the primary method in patients with stages I–III (100%, 100% and 36%, respectively), with postoperative radiotherapy and/or chemotherapy if indicated (adjuvant radiotherapy for patients in CS II–III, adjuvant chemotherapy according physician decision) Cytoreductive surgery in combination with preoperative or adjuvant chemotherapy was performed in only 24% of stage IV patients, whereas chemotherapy alone was used in 11 (33.3%). Other methods included chemotherapy in combination with palliative radiotherapy or best supportive care.

In patients undergoing neoadjuvant (*n* = 21) or palliative (*n* = 17) chemotherapy, multiple‐drug regimens were used: ADOC (doxorubicin, cisplatin, vincristine, cyclophosphamide), PE (cisplatin, etoposide), PAC (cisplatin, doxorubicin, cyclophosphamide) and PN (cisplatin, vinorelbine).

No complete responses (CR) were found within the group of patients treated with chemotherapy. In total, 21 partial responses (PR), 11 stable disease (DS) and two progressive disease (PD) as the best responses were noted. The data were not available in one patient receiving the PE chemotherapy regimen.

Table 2 presents the rates of different responses to systemic therapy (neoadjuvant and palliative chemotherapy combined).

**Table 2:** Response rates to initial and palliative chemotherapy (frontline)

Chemotherapy regimen^†^	Number of patients	CR	PR	SD	PD	ND
ADOC	19	‐	12 (63%)	5 (26%)	1	‐
PE	13	‐	7 (53%)	4 (30%)	‐	1
PAC	2	‐	1	‐	‐	‐
PN	3	‐	1	1	1	‐
CN	1	‐	‐	1	‐	‐

^†^ ADOC, doxorubicin 40 mg/m^2^ day 1 every 21 days; cisplatin 50 mg/m^2^ day 1 every 21 days; vincristine 0.6 mg/m^2^ day 3 every 21 days; cyclophosphamide 700 mg/m^2^ day 4 every 21 days.

PE, cisplatin 75 mg/m^2^ day 1 every 21 days; etoposide 100 mg/m^2^ day 1–3 every 21 days.

PAC, cisplatin 50 mg/m^2^ day 1 every 21 days; doxorubicin 50 mg/m^2^ day 1 every 21 days; cyclophosphamide 500 mg/m^2^ day 1 every 21 days.

PN, cisplatin 75 mg/m^2^ day 1 every 21 days; vinorelbine 30 mg/m^2^ day 1 and 8 every 21 days.

CN, carboplatin, 5 or 6 AUC every 21 days; vinorelbine 30 mg/m^2^ day 1 and 8 every 21 days.

CR, complete response; PR, partial response; SD, stable disease; PD, progressive disease; ND, lack of data.

The disease relapsed in 51 patients (27%), most often in stages III and IV (50% and 60%, respectively).

In total, 63 patients (33.5%) died, most often due to disease progression (*n* = 43; 68%).

### Overall survival

**Figure 2:**
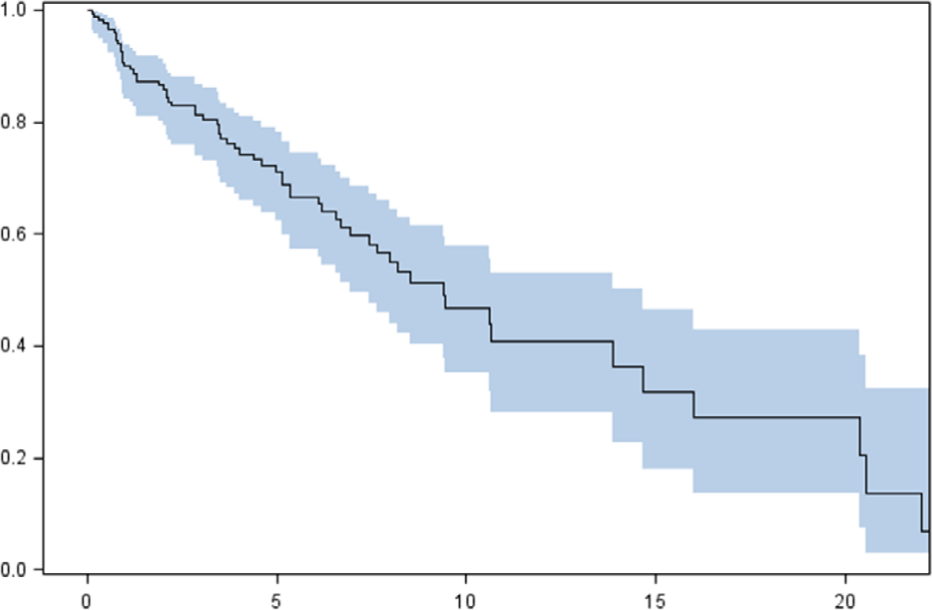
Cumulative overall survival probability in total population estimated by the Kaplan‐Meier method, with a 95% confidence interval (dashed line designates the median). Survival probability (%). Follow‐up (years). Median OS (total population): 9.4 years (95% confidence interval [CI] 6.9–14.6). Blue area – 95% confidence interval (CI).

The median OS in the analyzed population was 9.4 years (95% confidence interval [CI]: 6.9–14.6); 25% of patients survived more than 20 years and four months (upper quartile); and 75% of patients survived more than four years (lower quartile) (Fig 2).

**Figure 3:**
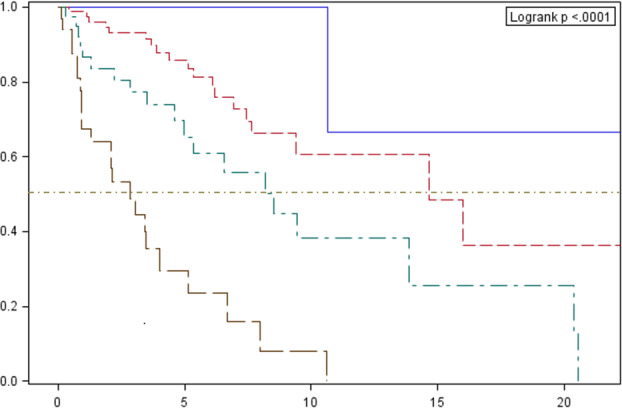
Cumulative overall survival probability according to Masaoka classification estimated by the Kaplan‐Meier method, with a 95% confidence interval (dashed line designates the median). Survival probability (%). Follow‐up (years). Clinical stage: I# II# III# IV. *P‐value for comparisons between curves: 0.2032 (1 vs. 2); 0.3230 (1 vs. 3); <0.0001 (1 vs. 4); 0.0323 (2 vs. 3); <0.0001 (2 vs. 4); 0.0132 (3 vs. 4).

**Figure 4:**
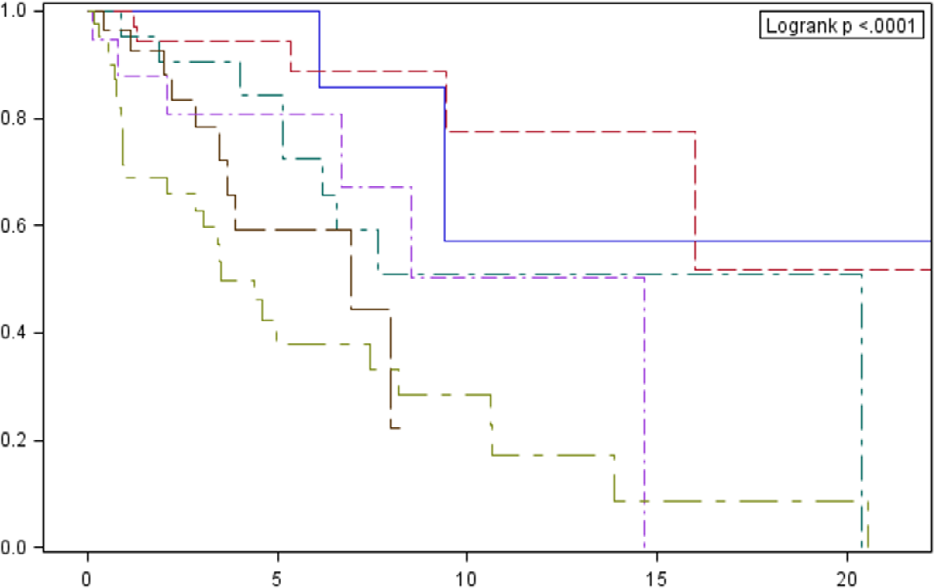
Cumulative overall survival probability in total population in relation to histological type according to WHO classification estimated by the Kaplan‐Meier method with a 95% confidence interval (the dashed line designates the median). Survival probability (%). Follow‐up (years). Histological type: A# AB# B1# B2# B3# carcinoma. *P‐value for comparison between curves: 0.3816 (A vs. AB); 0.9961 (A vs. B1); 0.461 (A vs. B2); 0.9103 (A vs. B3); 0.0002 (A vs. carcinoma); 0.2704 (AB vs. B1); 0.0125 (AB vs. B2); 0.0669 (AB vs. B3); <0.0001 (AB vs. carcinoma); 0.8875 (B1 vs. B2); 0.9990 (B1 vs. B3); 0.0072 (B1 vs. carcinoma); 0.9641 (B2 vs. B3); 0.0056 (B2 vs. carcinoma); 0.0004 (B3 vs. carcinoma).

The median OS in relation to clinical stage (22.6, 14.7, 8.5 and 3.5 years for stages I, II, III and IV, respectively) and histological type according to WHO are presented in Figs 3 and 4.

**Figure 5:**
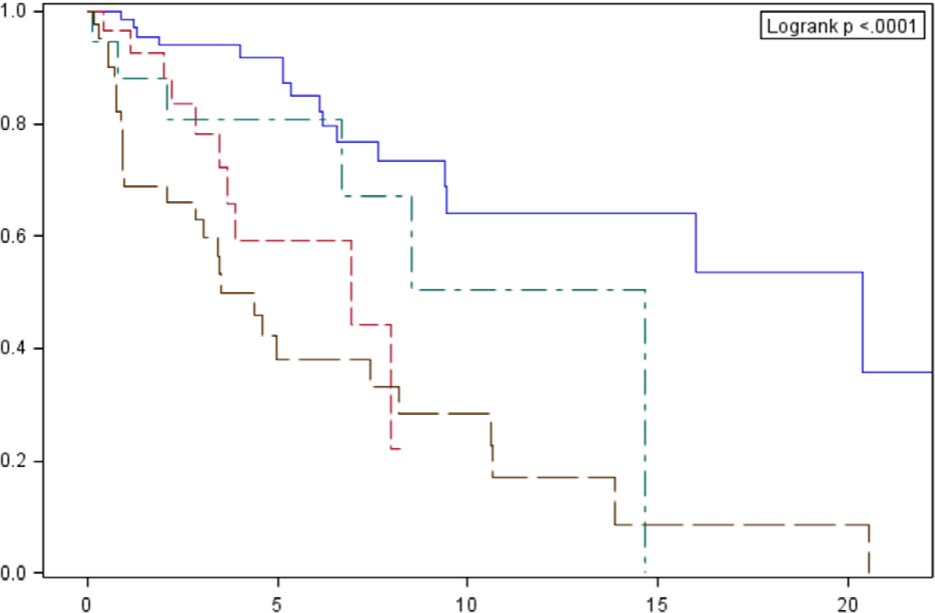
Cumulative overall survival probability among the total population, in relation to histological type according to the WHO classification estimated by the Kaplan‐Meier method, with a 95% confidence interval (dashed line designates the median) – combined analysis for types A, AB and B1. Survival probability (%). Follow‐up (years). Histological type: A, AB, B1# B2# B3# carcinoma.

In addition, due to the similar course of survival curves, patients diagnosed with thymomas A, AB and B1 were combined in one group. Significant differences in OS within this combined group were demonstrated in relation to patients with type B2 (*P* = 0.0005), B3 (*P* = 0.0030) and thymic carcinoma (*P* < 0.0001), with no statistically significant differences between B2 and B3 patients (Fig 5).

**Figure 6:**
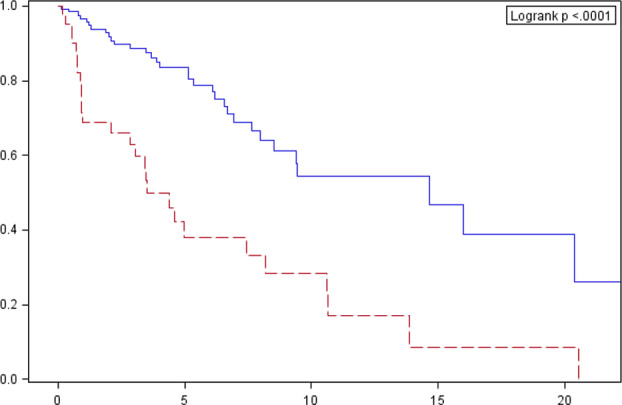
Cumulative overall survival probability estimated by the Kaplan‐Meier method in a group of patients diagnosed with thymic carcinoma, against the total population. Survival probability (%). Follow‐up (years). Histological type: Other than thymic carcinoma# Thymic carcinoma.

Patients with thymic carcinoma were characterized by the most unfavorable prognosis when compared with the total population. Survival curves are shown in Fig 6.

#### Overall survival: Prognostic factors

There was no statistically significant difference between OS for female and male patients (median 10.5 years vs. 8.2 years, respectively; *P* = 0.4255).

An analysis of patients with weight loss of 10% or more (*n* = 38; 20.3%) and patients without weight loss or reduction of <10% (*n* = 149; 79.7%) showed that significant weight loss (≥10%) is an independent negative prognostic factor. The median OS among patients without and with weight loss was 10.6 years and 2.1 years, respectively (*P* < 0.0001). The risk of death in patients with weight loss of ≥10% in a univariate Cox proportional hazard analysis was 5.57 (95% CI: 3.13–9.9; *P* < 0.0001).

The median OS among patients without comorbidities (*n* = 83; 43.9%) was 14 years and was longer than in patients with at least one concomitant disease (*n* = 105; 56.1%), for whom it was six years (*P* = 0.0008). The risk of death in a univariate Cox proportional hazard analysis was 2.41 (95% CI: 1.42–4.10; *P* = 0.0012).

There were no statistically significant differences between OS, depending on the occurrence of autoimmune diseases (*P* = 0.3387) or myasthenia gravis present (*P* = 0.8552) (data not shown).

Univariate logistic regression analysis, with LDH activity as an independent variable and survival as a dependent variable, indicated that the threshold LDH value with prognostic significance was 185 U/L, with 66 patients in the total population (35.5%) having a value ≥185 U/L. The risk of death was 2.81 (95% CI: 1.69–4.64). The median OS among patients with LDH activity <185 U/L and > 185 U/L was 10.6 years and 5.1 years, respectively (*P* < 0.0001).

Additionally, OS analysis was performed among the total population, depending on the WHO performance status and other laboratory and clinical parameters – white blood cell count (WBC), hemoglobin concentration, tumor size and lymph node involvement. The results of these analyses are summarized in Table 3.

**Table 3:** Survival parameters (OS), depending on the selected clinical variables

Clinical variable	Threshold values	Number of patients (%)	Median OS (years)	Hazard ratio (95% CI)	*P*‐value^†^
WBC count (g/L)	≤12.5	171 (91)	9.4	3.13 (1.52–6.45)	0.0011
	>12.5	17 (9)	3.5		
Hemoglobin concentration (g/dL)	≤9.5	13 (7.1)	12.1	3.75 (95% CI: 1.76–7.96	0.0006
	>9.5	175 (92.9)	9.5		
Baseline tumor size (cm)	<11	158 (83.2)	10.5	2.62 (95% CI 1.52–4.52)	0.0003
	≥11	30 (16.8)	4		
Lymph node involvement	Yes	24 (13)	2.8	nd	0.0001
	No	164 (87)	9.4		
WHO performance status	0	54 (28.7)	14.7	0.38 (0.19–0.73) (0 vs. 1)	*P* = 0.0219 (0 vs. 1)
	1	120 63.8)	6.9	0.12 (0.05–0.33) (0 vs. 2)	*P* < 0.0001 (0 vs. 2)
	2	10 5.3)	1.8	0.33 (0.15–0.74) (1 vs. 2)	

^†^ Calculated using logistic regression analysis of univariate Cox proportional hazard model.

#### Overall survival in clinical stage IV patients

In addition, an analysis was performed among patients with clinical stage IV. Weight loss and LDH activity (cutoff point <247 U/L) were significant prognostic factors in a group of 33 patients. There was no effect on OS of other analyzed parameters (performance status, presence of comorbidities). The median OS among patients with no weight loss was 3.5 years versus 11 months in patients with weight loss (*P* = 0.04). The median survival of patients with LDH activity equal to or less than 247 U/L was 3.5 years versus 10.8 months in the group of patients with LDH above 247 U/L (*P* = 0.04). There was no significant difference in OS between patients with sole pleural metastases (Masaoka IVA; *n* = 14) and patients with spread to other organs (Masaoka IVB; *n* = 19) (*P* = 0.16). However, it was shown that the presence of distant metastases is a negative prognostic factor for the total analyzed population (*P* < 0.001).

#### Radiotherapy

Although this publication does not focus on this issue, it is worth emphasizing the role of postoperative radiotherapy in the group of patients in stage II, according to Masaoka (99 patients). In total, 10.2% of patients did not undergo radiation therapy, which translated into an increased risk of death (*P* = 0.0374). The median survival among patients who did not undergo radiotherapy was seven years, while in the group of patients who received radiation therapy was 16 years.

#### Overall survival ‐ multivariate analysis

A multivariate analysis demonstrated that the independent negative risk factors in patients undergoing surgery were advanced clinical stage, weight loss, comorbidities, and increased LDH activity.

The highest risk of death was observed in patients in stage IV of the disease, according to Masaoka, and this was nearly 20 times higher than the risk of death in patients in stage I. The risk of death was also about 3.3 times higher in patients with weight loss and comorbidities.

## Recurrence‐free survival

A separate analysis of risk factors for recurrent disease was performed among patients who had undergone primary surgical treatment. The results are shown in Table 4.

**Table 4:** Univariate analysis of parameters potentially related to the risk of relapse in patients undergoing surgery

Parameter	Hazard ratio (95% CI)	*P*‐value
R1 to R0	1.23 (0.37–4.06)	0.7372
Male gender	0.796 (0.441–1.437)	0.448
Tumor size	1.088 (0.994–1.192)	0.0671
Postoperative radiotherapy	0.471 (0.253–0.880)	0.0182
Masaoka II vs. Masaoka I	1.311 (0.387–4.425)	0.6640
Masaoka III vs. Masaoka I	3.154 [0.869–11.494)	0.0807
Masaoka IV vs. Masaoka I	13.157 (3.413–50)	0.0002
Histological type B2 vs. A, AB, B1	3.077 (1.355–6.99)	0.0072
Histological type B3 vs. A, AB, B1	1.815 (0.668–4.926)	0.2420
Histological type carcinoma vs. A, AB, B1	4.566 (2.075–10.00)	0.0002
Weight loss	2.606 (0.998–6.802)	0.0504
Comorbidities	2.310 (1.257–4.246)	0.0070

## Discussion

TETs constitute a morphologically and clinically diverse group of rare anterior mediastinal tumors. Most of the studies published to date are retrospective, while only few studies were conducted prospectively with the aim being to assess the efficacy and safety of new systemic treatments among patients with advanced tumors.^16^, ^17^, ^18^, ^19^

The prognostic factors in the general population of patients diagnosed with primary TETs include clinical stage according to the Masaoka classification and surgical treatment (R0 versus R1 and R2).^9^, ^20^, ^21^ An analysis of the literature also indicates the prognostic value of histological type and tumor size.^22^, ^23^

This study is based on an analysis of 188 patients with median follow‐up of 11 years. The majority of patients were diagnosed and treated at the Department of Lung Cancer and Chest Tumors, Maria Skłodowska‐Curie National Research Institute of Oncology and at National Tuberculosis and Lung Diseases Research Institute in Poland. This allowed reliable data to be obtained, based on standardized diagnostic and therapeutic procedures (Table 5).

**Table 5:** Results of Cox proportional hazard analysis in the total population

Parameter	Hazard ratio (95% CI)	*P*‐value
Masaoka II vs. I	3.83 (0.49–29.76)	<0.0001
Masaoka III vs. I	6.18 (0.79–48.06)	
Masaoka IV vs. I	19.97 (2.47–161.25)	
Weight loss	3.35 (1.66–6.75)	<0.0001
Comorbidities	3.27 (1.75–6.08)	0.0011
LDH activity (an increase of 20 IU)	1.043 (1.019–1.067)	0.0002

The clinical stage is an established prognostic factor.^3^, ^6^, ^7^, ^9^, ^21^ The present study confirms that clinical stage according to the Masaoka classification is an independent prognostic factor. The highest percentage of deaths (69.7%) was recorded among patients in stage IV of the disease (*P* = 0.0001). The OS rate in this group of patients was significantly shorter, compared to that of patients in stages I, II and III (*P* < 0.001).

Several studies analyzed the prognostic value of the histological type of TETs.^3^, ^7^, ^21^ A correlation was found between the histological type of thymoma and the clinical stage. In patients with type A, AB and B1, according to the WHO classification, stages I or II at diagnosis were predominant, while in the majority of patients with type B3, stages III or IV were diagnosed (*P* < 0.001). Whilst there were differences in OS related to histological type, a multivariate analysis did not show an independent influence of histological type on prognosis. It was only shown that the histological type was an independent factor predicting the risk of relapse among patients with stages I and II (types B1, B2 and B3 vs. type AB) of the disease.^23^

In our analysis, a correlation was also found between the histological type and clinical stage. Clinical stages I or II were most frequently seen among patients with type A, AB and B1, while stages III or IV were the most common in thymic carcinomas patients. Histological type was shown to determine survival rates. Differences in OS were also noted when comparing patients (combined analysis) with thymomas type A, AB and B1 versus those with type B2 and B3 tumors. Our results are consistent with the reports of other authors.^23^, ^24^, ^25^, ^26^ However, in line with ITMIG observations, multivariate analysis did not show an independent prognostic value of the histological type in the studied population.^23^

In addition to the aforementioned prognostic factors, an analysis of other clinical parameters was performed in terms of their prognostic significance.

It was found that gender was not a prognostic factor, which is consistent with the observations of other authors, indicating a similar course of the disease among women and men.^9^, ^26^, ^27^

The study showed that increased LDH activity is an independent prognostic factor of OS, and the risk of death in patients with LDH activity >185 U/L was 2.81. A similar result was obtained by Wu *et al*. in the analysis of 90 patients diagnosed with stage III or IV thymic cancer (LDH 190 U/L – relative risk of death 2.7; *P* = 0.004)^28^ and Valdivia *et al*. who showed a very clear tendency for increase of LDH activity among patients with stage IV thymic carcinomas, according to the Masaoka classification.^10^ The studies involving patients with other cancers of the chest area also confirmed the prognostic value of increased LDH activity.^29^, ^30^

The present study also showed that WBC counts ≥12.5 G/L correlate with a worse prognosis (*P* = 0.0011). However, the results of univariate analysis were not confirmed in multivariate analysis. Murian *et al*. demonstrated a statistically significant correlation between neutrophil‐to‐lymphocyte ratio (NLR) and disease‐free survival (DFS) in patients stratified by TNM (*P* = 0.0043). A five‐year DFS rate in patients with low NLR compared to high NLR was 100% and 84% in stage I–II, according to TNM and 66% and 0% in stage III, respectively.^11^ Anemia was not shown to be an independent prognostic factor. No literature data related to either of these issues were found.

The studies published to date have shown that myasthenia gravis was significantly more common in women and in patients with histological types B2 and B3, according to the WHO. Multivariate analysis, however, did not show differences in OS in patients with myasthenia gravis, compared to patients without myasthenia (*P* = 0.956) .^9^, ^31^, ^32^ Furthermore, studies analyzing the presence of autoimmune diseases other than myasthenia gravis did not demonstrate their prognostic value (*P* = 0.18).^33^

Myasthenia gravis was found in 47 patients (25%) in the analyzed population, and other autoimmune diseases in 18 patients (9.6%). There were no differences in OS in both analyses (*P* = 0.85 and *P* = 0.33, for myasthenia gravis and other autoimmune diseases, respectively).

While the prognostic value of performance status was analyzed in a few publications that included general populations of patients with primary thymic tumors, the results of these observations are ambiguous.^34^, ^35^

In the present study, the analysis of the prognostic value of the WHO performance status showed statistically significant differences in the univariate analysis in whole population, as well as for patients diagnosed with thymic carcinoma. The worst prognosis was demonstrated in patients with performance status 2 (*P* < 0.001). Multivariate analysis did not confirm that performance status was an independent prognostic factor. This could be related to the heterogeneous distribution of subgroups – the assessed group comprised patients at stages I and II, undergoing radical surgery, which is the standard of treatment and determines the OS.

Although some reports regarding the prognostic value of weight loss in patients with TETs can be found, their results are inconclusive.^22^, ^36^ In the patients analyzed in the present study, it was shown that significant weight loss is an independent negative prognostic factor (*P* < 0.0001).

Significant comorbidities (eg, coronary artery disease, hypertension or chronic obstructive pulmonary disease) may influence outcomes significantly. The problem particularly applies to elderly patients.^37^, ^38^, ^39^, ^40^

In the present study, concomitant diseases were found in 56% of the patients, predominantly hypertension and ischemic heart disease. Internal diseases were shown to be an independent prognostic factor for OS, although the value of this observation is uncertain, due to the methodological limitations of the analysis. No literature data concerning the effect of comorbidities on OS in the general population of patients with thymic tumors were found.

In several publications, the prognostic value of the thymic tumor diameter was postulated.^9^, ^22^, ^27^, ^41^

In this study, the prognostic value of tumor diameter was analyzed for both the general population and patients undergoing surgical treatment (data not shown). It is worth emphasizing that the limit values used, ie, 11 cm in the general population and 10 cm in patients primarily undergoing surgical treatment, were larger than those found in previous publications. This may result from the fact that in some cases, the tumor size was assessed based on CT, which has lower reliability than the description of the material obtained during thymectomy. The risk of death demonstrated in patients with a tumor size ≥11 cm was higher (HR = 2.62; 95% CI: 1.52–4.52).

The majority of analyses of the prognostic role of the metastatic site included patients with thymic carcinoma. However, their results are contradictory.^42^, ^43^, ^44^, ^45^, ^46^ The result of no differences in OS among patients in the analyzed group with stage IVB tumors, compared to IVA, may be related to a small sample size. In addition, the majority of patients in both subgroups were diagnosed with carcinoma or B3 thymoma (histological types with similar and the worst prognosis). The extrapulmonary metastases were shown to be a negative prognostic factor for the entire analyzed group (*P* < 0.001). This observation confirms the independent prognostic value of stage IV, according to Masaoka, and indicates to a group of patients with the least favorable disease course (patients with extrapulmonary metastases).

Lymph node metastases are found in a negligible percentage of patients with thymomas (less than 2%), but many authors emphasize the prognostic value of this factor, especially in thymic carcinomas.^47^, ^48^, ^49^

In a univariate analysis of the total evaluated population, the lymph node metastases were a negative prognostic factor (*P* < 0.001). Multivariate analysis, however, did not confirm this observation.

TETs constitute a group of tumors with a fairly good prognosis, but varied clinical presentation,with the worst outcomes in thymic carcinomas patients.^21^ When interpreting the prognostic data, we must take into consideration that only 50%–70% of all the patients actually die from TET progression, because causes of death include autoimmune diseases and nonrelated disorders.

Clinical stage was the most important and independent prognostic factor. A closely related parameter was the possibility of complete surgical resection, which is a well‐established standard of care. R0 resection was a favorable prognostic factor in the study group. Some additional parameters‐ histological type, lymph node involvement, WHO performance status, anemia, leucocytosis, LDH level, concomitant diseases and weight loss can be risk factor for disease recurrence. As the analysis was done retrospectively‐ based on medical records, the results may be of limited value. As mentioned previously, an independent radiological and histological reassessment was not performed. The multidisciplinary cooperation of a thoracic surgeon, medical oncologist, radiation oncologist, pathologist and neurologist is of fundamental importance in the diagnostic and therapeutic process.

In conclusion, TETs are a group of neoplasms with a fairly good prognosis, which vary in their clinical presentation. Disease dissemination is an independent most significant prognostic factor. Surgery is the fundamental treatment method for primary thymic tumors, and complete resection is an important prognostic factor. Patients with stage II disease benefit from postoperative radiotherapy. Among laboratory factors, low hemoglobin concentration (9.5 g/dL or less), high WBC count (above 12.5 g/L) and high LDH activity (above 185 U/L) have a negative impact on survival parameters. Comorbidities and weight loss are negative prognostic factors in the general population. Tumor size (diameter over 11 cm) and metastases in lymph nodes are negative prognostic factors for the total analyzed population.

**Disclosure**

The authors declare that there are no conflicts of interest. The research did not receive any specific grant from funding agencies in the public, commercial, or not‐for‐profit sectors.
